# Explainable artificial intelligence for heart rate variability in ECG signal

**DOI:** 10.1049/htl.2020.0033

**Published:** 2020-12-09

**Authors:** Sanjana K., Sowmya V., Gopalakrishnan E.A., Soman K.P.

**Affiliations:** Center for Computational Engineering and Networking, Amrita School of Engineering, Amrita Vishwa Vidyapeetham, Coimbatore, Tamilnadu, India

**Keywords:** signal classification, learning (artificial intelligence), cardiovascular system, diseases, electrocardiography, medical signal processing, convolutional neural nets, tachycardia disease, deep learning model, atrial fibrillation, ventricular fibrillation, sinus tachycardia, deep learning models, CU-ventricular tachycardia data, cardiac diseases, deep learning architectures, ECG signal, electrocardiogram signal, MIT-BIH malignant ventricular ectopy database, RCNN model

## Abstract

Electrocardiogram (ECG) signal is one of the most reliable methods to analyse the cardiovascular system. In the literature, there are different deep learning architectures proposed to detect various types of tachycardia diseases, such as atrial fibrillation, ventricular fibrillation, and sinus tachycardia. Even though all types of tachycardia diseases have fast beat rhythm as the common characteristic feature, existing deep learning architectures are trained with the corresponding disease-specific features. Most of the proposed works lack the interpretation and understanding of the results obtained. Hence, the objective of this letter is to explore the features learned by the deep learning models. For the detection of the different types of tachycardia diseases, the authors used a transfer learning approach. In this method, the model is trained with one of the tachycardia diseases called atrial fibrillation and tested with other tachycardia diseases, such as ventricular fibrillation and sinus tachycardia. The analysis was done using different deep learning models, such as RNN, LSTM, GRU, CNN, and RSCNN. RNN achieved an accuracy of 96.47% for atrial fibrillation data set, 90.88% accuracy for CU-ventricular tachycardia data set, and also achieved an accuracy of 94.71, and 94.18% for MIT-BIH malignant ventricular ectopy database for ECG lead I and lead II, respectively. The RNN model could only achieve an accuracy of 23.73% for the sinus tachycardia data set. A similar trend is shown by other models. From the analysis, it was evident that even though tachycardia diseases have fast beat rhythm as their common feature, the model was not able to detect different types of tachycardia diseases. The deep learning model could only detect atrial fibrillation and ventricular fibrillation and failed in the case of sinus tachycardia. From the analysis, they were able to interpret that, along with the fast beat rhythm, the model has learned the absence of P-wave which is a common feature for ventricular fibrillation and atrial fibrillation but sinus tachycardia disease has an upright positive P-wave. The time-based analysis is conducted to find the time complexity of the models. The analysis conveyed that RNN and RSCNN models could achieve better performance with lesser time complexity.

## 1 Introduction

Cardiovascular disease (CVD) is one that affects the heart and blood vessels. The CVDs include coronary heart disease, rheumatic heart disease, etc. [1]. The risk of the CVDs increases due to blood clots that are caused by the build-up of fat deposits in the coronary arteries. According to the study conducted by WHO, an estimated 17.9 million people died due to CVDs in 2016, i.e. 31% of all deaths worldwide [2]. The CVD in a broader sense can be categorised into electrical disorder, circulatory disorder, and structural disorder [3]. The electrical disorder is caused due to the malfunction of the electrical system that synchronises the heartbeat (e.g. arrhythmia). The circulatory disorder is caused due to the high blood pressure and block in the coronary artery (e.g. stroke or heart attack). The structural disorder is caused due to the damage in the heart muscle or heart valves (e.g. cardiomyopathy).

Most of the people might have experienced irregular heart rhythms at some point in their life. Arrhythmia is developed when there is an abnormality in electrical impulse formation or transformation or abnormality in both [4]. Some of the arrhythmias are a threat to life [3]. When the heart beats are slower than the normal heart rate (<50 bpm) it is called bradycardia or bradyarrhythmia. In such cases, the blood pressure cannot be controlled and the patient will faint which leads to death. Similarly, when the heart beats faster than the normal heart rate (>100 bpm) it is called tachycardia or tachyarrhythmia. This may lead to pass out and sudden death [5]. As arrhythmias are one of the main causes of mortality, detection of the arrhythmias at the early stage has acquired great importance in recent years. Tachycardia and bradycardia can be classified into different types based on their origin. The different types of tachycardia include ventricular fibrillation (VF), long QT syndrome, premature ventricular contractions, atrial flutter (AFL), supraventricular tachycardia (SVT), atrial fibrillation (AF), sinus tachycardia (ST) and Wolff–Parkinson–White syndrome [6]. The different types of bradycardia include sinus bradycardia (SB), sinus pause, or sinus arrest, sick sinus syndrome [7, 8].

**Fig. 1:**
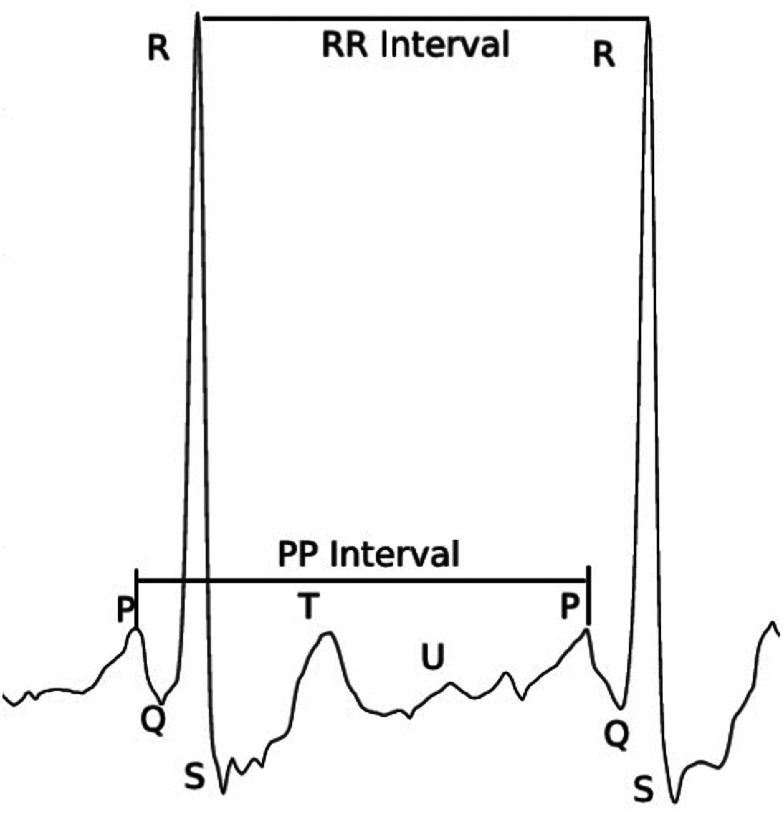
Normal ECG signal [12]

As the risk of heart disease is high, the detection of disease must be accurate. Different techniques prevailed in the detection of coronary heart diseases. Some of the techniques are the electrocardiogram (ECG), Holter monitoring, echocardiogram, stress test, cardiac catheterisation, cardiac computerised tomography (CT) scan, and cardiac magnetic resonance imaging (MRI) [9]. Among the above-mentioned techniques, ECG based analysis is the most commonly used practice to diagnose cardiac disease. An ECG signal is a record of electrical communication of the heart. ECG signal monitoring is a non-invasive technique. ECG signals are recorded by placing small electrodes in the legs, arms, and chest. Cardiac disease is detected through the analyses of variation in the morphology of the ECG signal. The characteristic feature of a normal ECG signal during one cardiac cycle is the P-wave followed by the QRS complex continued by a T-wave [10, 11]. The sample of the normal ECG signal is shown in Fig. 1 [12].

The intervals between the waves P-QRS-T varies when the person is affected by the disease. The variation of the ECG signal based on the characteristic shape and interval helps the experts in disease diagnosis. However, analysis of the ECG is a complex procedure as the experts should consider various factors, such as age, gender, previous health condition, etc. Along with this, the number of patients a doctor would see during a day is also very high and so, it is also prone to error. To make ease of this task, an automatic expert system to diagnose cardiac disease is preferable.

Automation in the expert system aims to make an intelligent system that can automatically detect disease. Advancement in the field of artificial intelligence made automation in expert systems possible [12]. Conventional methods require feature extraction that is specific for the disease from the raw signal. The model should be fed with optimal data. The model trained with less amount of data shows poor performances due to overfitting. A deep learning-based model in contradiction to the machine learning model can learn the required features by itself [13]. In the case of cardiac disease, each of the tachycardia and bradycardia disease contains features, which have disease-specific variability. Fast beating rhythm is common for all the tachycardia diseases. In the case of the atrial fibrillation (AF) and VF, the specific feature that makes them distinct from another tachycardia disease, i.e. ST data set is the absence of the P-wave. The VF has the presence of the fibrillatory waves in the QRS baseline [6–8]. The deep learning model is expected to study different disease-specific features for the detection of different diseases.

Arrhythmia is the most dangerous cardiac disease which can be life-threatening because of its abnormal heart rate. Various studies have been conducted in this field for the detection of different types of arrhythmia, which is generally classified as tachycardia and bradycardia. Acharya *et al.* [14] proposed an automatic system to classify different segments of an ECG signal. The proposed method used a convolution neural network (CNN) that classified the data into four classes, namely AF, VF, atrial flutter, and normal. The model was able to achieve an accuracy of 92.50%, sensitivity of 98.09%, and specificity of 93.13%. Wang *et al.* [15] performed a novel short-time multi-fractional approach to classify AF, VF, and ventricular tachycardia (VT). With a fuzzy Kohonen classifier, the proposed method achieved an accuracy higher than 97%. Martis *et al.* [16] used the discrete cosine transform together with independent component analysis (ICA) as a dimensionality reduction approach. K-nearest neighbour algorithm based classifier has been used to classify diseases, such as AF and atrial flutter from normal ECG beats. The method acquired an accuracy of 99.45%. A higher-order spectra method was proposed by Martis *et al.* [17] for rectifying the problem due to high nonlinearity in the ECG signal and compared two higher-order methods for classification of the three diseases namely AF, atrial flutter, and normal. This method obtained an accuracy of 97.65% and a predictive value of 99.53%. Khadra *et al.* [18] used higher-order bi-spectral analysis for classification of arrhythmias, such as AF, VF, and VT with respect to normal (NR) ECG. Sensitivity values of 91.7, 81.8, 83.3, and 100% were obtained for VF, VT, AF, and NR, respectively. Li *et al.* [19] used a support vector machine-based method for the classification of VF and VT. The proposed method achieved an accuracy of 96.3%. Assodiky *et al.* [20], Isin and Ozdalili [21], and Alfaras *et al.* [22] proposed methods for the automatic detection and classification of the ECG signals. Gee *et al.* [23] proposed explainable deep learning using two-dimensional (2D) time-series ECG data. The present work concentrates mainly on interpreting the features learned by the model trained with the 1D time-series ECG data. Andreotti *et al.* [24] compared the performances of feature-based classifiers and CNN for the detection of AF disease. The proposed method achieved an F1 score of 0.79 for the feature-based classifier. The CNN gained an F1 score of 0.83 for the test set. Andersen *et al.* [25] proposed a deep learning method which is a combination of the CNN and the recurrent neural network (RNN) for AF detection in the long-term ECG signal. The proposed method achieved a specificity of 0.98 and a sensitivity of 0.86 for the unseen data. The proposed method requires much less time to analyse the 24 h ECG signal. Hannun *et al.* [26] proposed a deep learning method to classify the 12 different rhythms, such as AF, NSR (normal), noise, sudden Brady response, bigeminy, AFL (atrial flutter), EAR (bunny ear pattern), IVR (accelerated idioventricular rhythm), Wenckebach, trigeminy, SVT, VT. The proposed method gained a score of 0.97 for the area under the curve (AUC). The proposed method also acquired an F1_score of 0.837 for the classification of different types of cardiac rhythms. Shashikumar *et al*. [27] proposed a combination of a convolutional neural network and RNN based approach for the detection of paroxysmal AF. The convolutional neural network is fed with sequential segments of a signal as the time–frequency domain represents signal images. The output of the deep learning model is the features of the images. The features extracted are fed to RNN for the detection of AF. The proposed approach achieved an AUC of 0.94.

Sujadevi *et al.* [28] proposed RNN-based AF detection. The work made use of architectures, such as an RNN, long-short term memory (LSTM), and gated recurrent unit (GRU) for the real-time detection of AF which gained accuracies of 95, 100, and 100%, respectively. Kiranyaz *et al.* [29] proposed a 1D CNN-based adaptive method for individual specific ECG signal classification. The method was able to show reliable performance in the classification of ventricular ectopic beats and supraventricular ectopic beats. Kachuee *et al.* [30] used deep learning architecture for the classification of five different classes of arrhythmia and the approach gained an accuracy of 93.4%. Further, the authors have used the transfer learning approach because of the less availability of data. Transferred knowledge from the classification of arrhythmia is used to classify ECG signals with and without myocardial infraction with an accuracy of 95.9%. Gopika *et al.* [31] further showed an improved accuracy from 95.9 to 99% using the features proposed by Kachuee *et al.* [30].

From the literature, it is evident that there are various approaches used for the efficient classification of different types of tachycardia diseases. The different types of tachycardia diseases are AF, VF, and ST (AF, VF, and ST), which have the fast beat rhythm as a common feature. In the previous works, even though AF, VF, and ST have a common feature, the models are trained with disease-specific ECG signals for detection of the above mentioned different types of tachycardia diseases. In most cases, interpretation for the detection by the respective models is also missing. Hence the present work establishes the concept of explainable artificial intelligence (AI). Explainable AI is the field that has gained more popularity recently [32]. The interpretation and understanding which can be made out of the analysis of deep learning models are coined as explainable AI [32]. It tries to interpret the reason for the decision made in the black box of neurons. This interpretability helps to improve performance in various fields of AI. In the disease classification problem, we may not exactly know what the model learns. In the case of tachycardia disease, it is expected to learn the fast beat rhythm. The different types of tachycardia diseases, such as AF and VF, which do not contain specific P-wave segments are different from ST which have distinct P-wave segments. Other features in the ECG signal that make AF different from the other tachycardia data set is the presence of the fibrillatory waves in the baseline of the QRS complex. VF also has the fibrillatory waves in the baseline of ECG. However, more commonly, the ECG of VF is irregular and all segments (P, QRS, and ST) are distorted. One distinct feature used for the identification of ST is the presence of positive upright P-wave before the QRS complex. Other segments, such as QRS and ST have normal morphology [33, 34, 35]. The objective of the present work is to explore and analyse the features that the model has learned for the detection of tachycardia diseases. To explore our objective, state-of-art architectures of deep learning, such as LSTM, RNN, GRU, CNN, and residual skip CNN (RSCNN) [28, 30] are implemented in our present work. We considered different types of tachycardia diseases, such as AF, VF, and ST, which have fast beat rhythm as the common characteristic feature is used for the evaluation. To achieve this objective, we use the concept of transfer learning. In our present approach, the model trained with one of the tachycardia diseases is tested to detect other different types of tachycardia diseases unseen by the model during training.

## 2 Data set description

In this work, ECG signal data sets that are publicly available in the PhysioNet database [36] are used. Data set for AF disease is taken from the AF classification 2017 PhysioNet CinC challenge which is referred to as the tachycardia data set one (AF: TD1). The VF disease data set which is referred to as the tachycardia data set two (VF: TD2). This data set is retrieved from two sources namely Creighton University ventricular tachyarrhythmia (VF: TD2-A) and MIT-BIH ventricular ectopy (VF: TD2-B). MIT ventricular ectopy data set has ECG signals collected from two leads. The data set for ST (ST: TD3) is taken from the MIT-BIH arrhythmia database. The number of records of raw ECG signal and the corresponding number of samples based on feature extraction for all the above-mentioned data sets is presented in Table 1.

**Table 1:** Data set description

Data set	Number of records of raw ECG signal	Number of feature extracted samples
atrial fibrillation (AF: TD1) (AF data set CINC challenge 2017)	8528	64,767
ventricular fibrillation (VF: TD2-A) (Creighton University Ventricular Tachyarrhythmia Database)	33	1426
sinus tachycardia (ST: TD3) (MIT-BIH arrhythmia database of PhysioNet)	59	118
ventricular fibrillation (VF: TD2-B) (MIT-BIH malignant ventricular ectopy database)	22	945

The PhysioNet provides an open-source tool kit for the extraction of heart rate variability (HRV) features. Joseph *et al.* [36] in the background study proposed that for small segment signals which are of duration <15 min, time-domain features and frequency-domain features are suitable. The AF-TD1 data set has each signal varying in the time duration from 30 to 60 s. Thus, as mentioned in the PhysioNet tool kit, the time and frequency domain features are considered. The work proposed by Andreotti *et al.* [24] motivated us to include the nonlinear features and signal quality features. Hence, in the proposed work, the time-domain features, frequency-domain features, and nonlinear features along with the signal quality indices are included to formulate a 169 dimension feature vector [37].

Each sample is a feature vector with a dimension of 169 [24]. This feature vector contains information related to HRV indices and signal quality indices. The HRV indicates the changes in the heart beats per minute. The time-domain features give the fluctuations observed in the HRV over an interval of time. The time intervals may range from 2 min to 24 h. The frequency-domain features give the energy information of the ECG signal. The nonlinear features indicate the complexity and nonlinearity within interbeat intervals of the ECG signal. Signal quality indices represent the segment-wise features of the ECG signal [37]. Signals are separated as segments of 10 s with an overlap of 50% for constructing feature extracted samples [24] so that no pieces of information are lost.

The deep learning algorithms may perform better with the raw ECG signals. However, the deep learning algorithm trained with the handcrafted features performs better than the raw ECG signals [30]. However, the main objective of the present work is to interpret the features learned by the model in association with the abnormality due to the variation in heart beat rhythms. The main idea of extracting the HRV features is to make the interpretation of features learned by the model easy and clear. Feeding the raw signal and interpreting will just give vague ideas about the model performance. The contribution of each feature to accurately identify the normal and abnormal cases makes it easy to interpret the model.

The feature-based analysis rather than feeding the raw signal directly to the deep learning architectures is chosen based on the work proposed by Kachuee *et al.* [30]. One other advantage of using feature-based segments is that we can quantify the influence of the features for the results obtained. The use of these feature extracted samples reduces the high computational requirement for the deep learning approach. This gave the motivation to make use of the feature extracted segments.

## 3 Methodology

**Fig. 2:**
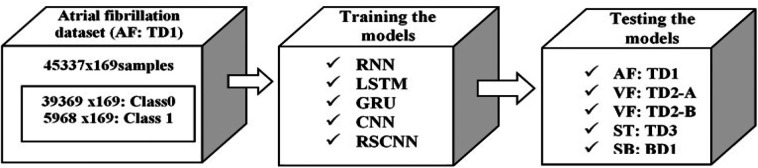
Proposed methodology for the detection of tachycardia diseases and for the interpretation of features learned by the model

The main objective of the present work is to interpret the features learned by the deep learning models for cardiac disease detection using ECG signals. In order to meet the objective, we consider one of the classes of cardiac diseases called tachycardia. The tachycardia contains fast beat rhythm as one of the main characteristic features of an ECG signal. The different types of tachycardia diseases are AF (AF: TD1), VF (VF: TD2), and ST (ST: TD3). As AF: TD1, VF: TD2, and ST: TD3 are the types of tachycardia diseases, ECG signals have fast beat rhythm as the common feature. Therefore, the deep learning model was trained with one of the tachycardia diseases called AF: TD1 and tested with the rest two types of tachycardia called VF: TD2 and ST: TD3. The VF: TD2 and ST: TD3 are unseen data sets by the model. This approach aids to interpret the common characteristic feature of ECG signals corresponding to different types of tachycardia diseases learned by the model. The overall workflow of the methodology for the interpretation of the features learned by the model is shown in Fig. 2. The proposed method consists of the following steps. Initially, the models are trained with the AF data set. The AF data set contains both abnormal and normal cases. Then the trained models using AF data set are tested with other tachycardia data sets, such as AF (AF: TD1), VF (VF: TD2-A and VF: TD2-B), and ST (ST: TD3) separately. The state-of-the-art deep learning architectures implemented in the present work are RNN, LSTM, GRU, CNN, and RSCNN [28–30].

### 3.1 Architecture details

The benchmarks deep learning architectures, such as RNN, LSTM, GRU, CNN, and RSCNN [28–30] are contemplated for the study. The details about RNNs are given in Table 2. The input layer of each model is modified to 169 × 1, as the input signal, has a feature vector of size 169 × 1. RNNs considered being RNN, LSTM, GRU, which have one hidden layer with 64 units. The second layer (output layer) is dense with the number of neurons the same as the number of classes considered. AF data set (AF: TD1) which is considered for training have two classes, i.e. the one without AF (normal: Class 0) and the other with AF (abnormal: Class 1). So, the final dense layer consists of two neurons with a sigmoid activation function.

**Table 2:** Architecture details of RNN, CNN, LSTM networks

Architecture	RNN	LSTM	GRU
input layer	169 × 1	169 × 1	169 × 1
recurrent layer	RNN	LSTM	GRU
output layer	dense layer (sigmoid)	dense layer (sigmoid)	dense layer (sigmoid)

**Fig. 3:**
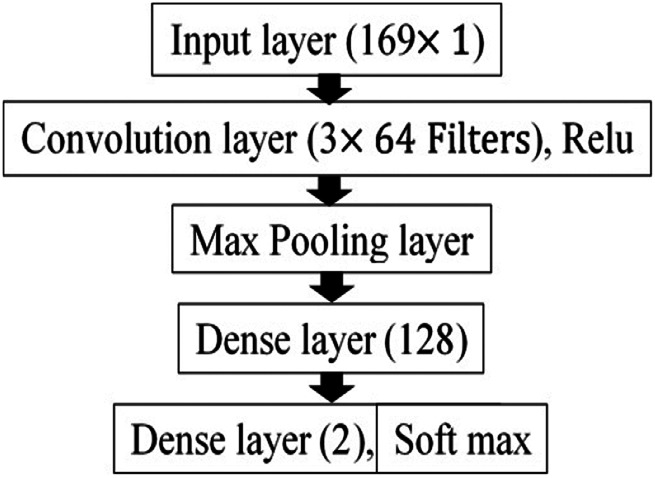
1D CNN

The number of learnable parameters varies according to the chosen architecture of the model. For the RNNs, the computation of the number of learnable parameters is given by

(1) $${\rm paramRNNs} = f \times \lsqb ns\lpar ns + i\rpar + ns\rsqb \eqno\lpar 1\rpar $$

where *f* is the number of fully connected neural networks, *ns* is the number of neurons in the hidden layer and *i* is the input size. In the case of RNN, the number of fully connected neural networks is 1, for GRU it is 3 and for LSTM it is 4. For dense layers, the number of the learnable parameters is computed by

(2) $${\rm paramDense} = \lsqb ns \times i + b\rsqb \eqno\lpar 2\rpar $$

where *b* is the bias. The details of the CNN model are shown in Fig. 3. The CNN model contains a convolution layer with 64 filters of size 3 with stride 1. This convolution layer is accompanied by ReLU (rectified linear unit) activation function. The output from the convolution layer is mapped into the nonlinear output using the activation function for avoiding the vanishing gradient problem. The model also contains two dense layers: one with 128 neurons and other with 2 neurons, which serve as the output layer with a soft-max activation function.

The design of the CNN architecture proposed by Swapna *et al.* [38] is used in the present work. In [38], the complexity of the model is validated in terms of the number of learnable parameters. The complexity along with model performance is taken into consideration to fix the number of neurons in the hidden layer. An increase in the number of neurons may increase the model performance, but along with it, there will be an increase in the number of learnable parameters. The reduction in the number of neurons may decrease performance [38]. Since better performance with lesser complexity is always appreciated, we fixed the CNN model with a single layer of 64 neurons.

The number of learnable parameters of the CNN is given by

(3) $${\rm paramCNNs} = nf \times fs + b\eqno\lpar 3\rpar $$

where *nf* is the number of filters, *fs* is the filter size and *b* is the bias.

**Fig. 4:**
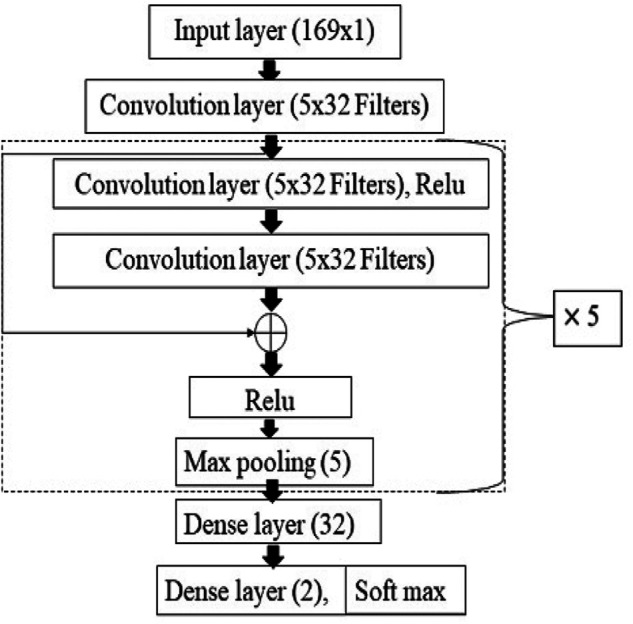
RSCNN

The details of the RSCNN architecture are shown in Fig. 4. RSCNN contains 13 weighted layers, which include 11 convolution layers and 2 dense layers. The first layer is the input layer of size 169 × 1, which is same as the size of the feature vector. Convolution layer has 32 filters with 3 as the filter size in each layer. The network has residual blocks. The residual blocks contain two convolution layers with the ReLU activation function. Succeeded by a max-pooling layer for the dimensionality reduction, a skip connection is also included in the residual block. The skip connection takes care of all the information to be carried without any loss from the first convolution layer to the final dense layer, as shown in Fig. 4, these residual blocks are repeated five times. The dense layer of 32 neurons is included after the residual blocks. The final output layer is a dense layer with 2 neurons with a soft-max activation function. The number of learnable parameters computed for all the benchmark architectures is tabulated in Table 3.

**Table 3:** Number of learnable parameters computed for RNN, LST M, GRU, CNN, and RSCNN

Architectures	Number of learnable parameters
RNN	15,170
LSTM	60,290
GRU	45,250
CNN	696,962
RSCNN	54,914

### 3.2 Training and testing

All the benchmark deep learning architecture (RNN, LSTM, GRU, CNN, and RSCNN) are trained with 70% of AF data (AF: TD1) for 1000 epochs with the batch size 1000 samples for each architecture. The models are tested with 30% of AF data and also with other tachycardia data sets, such as VF (VF: TD2-A and VF: TD2-B) and ST data (ST: TD3).

## 4 Result and analysis

Accuracy, sensitivity, F1 score, and specificity are the evaluation metric used for the performance assessment of all the deep learning architectures implemented in this work. In the biomedical field, the sensitivity score has very high importance. Let's consider the condition of disease as a positive case and the normal as a negative case from the medical perspective. Hence, sensitivity is the measure of the ratio of actual positive detected as positive. Since the disease unidentified is a threat to life, the sensitivity score is considered for the evaluation of the disease detection along with the accuracy. Specificity measures the effectiveness of the model to detect the normal cases. The measure of combined precision and sensitivity score is incorporated into the F1 score. The confusion matrix gives the exact idea about the number of samples that are correctly classified and miss classified. The four parameters used for the analysis of the confusion matrix are true positive (TP), false positive (FP), true negative (TN) and false negative (FN). TP represents the number of samples that are positive and predicted correctly as positive. FP represents the number of samples that are negative and predicted wrongly as positive. TN represents the number of samples that are negative and predicted correctly as negative. FN represents the number of samples which is positive and predicted wrongly as negative. The main purpose of the present work is to determine whether the model trained on one type of tachycardia can detect other types of tachycardia diseases. This is performed in order to interpret the common characteristics of ECG signals that are affected by different types of tachycardia diseases learned by the trained model. Hence, the model trained on AF: TD1 data set is tested with AF: TD1, VF: TD2-A, VF: TD2-B, and ST: TD3.

**Fig. 5:**
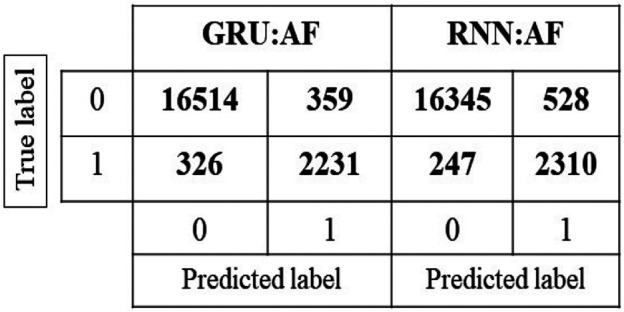
Confusion matrix for GRU and RNN model tested with AF: TD1 data

In AF data (AF: TD1), a total of 19,430 samples were tested in which 16,873 are class 0 and 2557 are class 1. For the normal class, the RSCNN model gained an accuracy of 97.92%, which is higher than other models and RNN gained an accuracy of 90.34%, which is higher than other models for abnormal class. While considering the average accuracy, including both classes, the GRU model has performed better than other models with an accuracy of 96.47%. While considering the sensitivity score for the abnormal class, RNN has gained a score of 0.90 which is higher than other models. Thus, for AF: TD1 RNN has performed better than other models in detecting the abnormal class. The accuracy score in percentage, sensitivity, F1 score, and specificity for the models tested with AF (AF: TD1) for class 0 and class 1 is given in Tables 4 and 5. Class 0 represents the normal class and class 1 represents the AF (abnormal class). The confusion matrix for the GRU and RNN model is shown in Fig. 5. The diagonal elements in the figure represent the TP and TN number of samples.

**Table 4:** Experimental results for AF CINC 2017 data set class 0 (AF: TD1 class 0)

	LSTM	GRU	RNN	CNN	RSCNN
accuracy, %	95.77	97.87	96.87	97.13	97.92
sensitivity	0.96	0.98	0.97	0.98	0.98
F1_score	0.97	0.98	0.98	0.97	0.98
specificity	0.957	0.96	0.96	0.97	0.97

**Table 5:** Experimental results for AF CINC 2017 data set class 1 (AF: TD1 class 1)

	LSTM	GRU	RNN	CNN	RSCNN
accuracy, %	88.03	87.25	90.34	78.14	83.93
sensitivity	0.88	0.87	0.90	0.78	0.84
F1_score	0.82	0.87	0.86	0.81	0.86
specificity	0.88	0.90	0.90	0.78	0.83

**Fig. 6:**
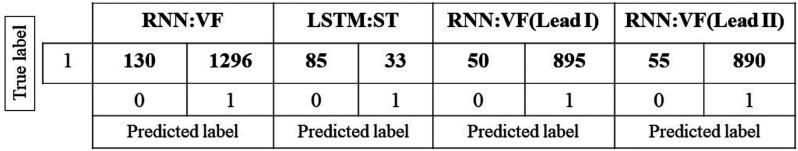
Confusion matrix for RNN model tested with VF: TD2-A, LSTM model tested with ST: TD3 data, RNN model tested with VF: TD2-B data for lead I and lead II

In the case of VF, CU-VT data set (VF: TD2-A) is used for the evaluation. The models are tested using 1426 samples of tachycardia data. The RNN has acquired a percentage accuracy of 90.34%, which is higher than other models. Analysing the sensitivity score of these models, we can understand that RNN has a better score of 0.91 than other models. The accuracy score in percentage, sensitivity, F1 score, and specificity for the models tested with VF (VF: TD2-A) is given in Table 6. The confusion matrix for the RNN model which is tested with VF: TD2-A is shown in Fig. 6. From the figure, it is clear that the number of TP samples is 1296.

**Table 6:** Experimental results for CU-VT data set (VF: TD2-A)

	LSTM	GRU	RNN	CNN	RSCNN
accuracy, %	82.75	77.84	90.88	74.89	68.93
sensitivity	0.83	0.78	0.91	0.75	0.69
F1_score	0.91	0.88	0.95	0.86	0.82
specificity	0.83	0.77	0.90	0.75	0.68

For the validation of the above result, the second data set of VF disease VF: TD2-B from the MIT-BIH malignant ventricular ectopy database was taken. The evaluation metrics of the models tested with VF: TD2-B for lead I and lead II ECG signals are given in Tables 7 and 8, respectively. In this data set, 945 samples of VF: TD2-B were tested on different models, such as LSTM, GRU, RNN, CNN, and RSCNN. Among these, RNN has shown better performance with an accuracy of 94.71% for the lead I ECG signal of VF: TD2-B. The RNN model has gained the best sensitivity score of 0.95 when compared with other models. The RNN has shown the same trend for VF: TD2-B lead II data with an accuracy of 94.18% and a sensitivity score of 0.94. From the result acquired from VF: TD2-A and VF: TD2-B data sets, it is clear that RNN had performed well for the detection of VF disease. The confusion matrix of the RNN model of VF: TD2-B for the lead I and lead II is shown in Fig. 6. It shows that TP samples are 895 and 890 for the lead I and lead II, respectively.

**Table 7:** Experimental results for MIT-BIH malignant ventricular ectopy database (VF: TD2-B-lead I)

	LSTM	GRU	RNN	CNN	RSCNN
accuracy, %	92.17	90.69	94.71	88.67	76.61
sensitivity	0.92	0.91	0.95	0.89	0.77
F1_score	0.96	0.95	0.97	0.94	0.87
specificity	0.92	0.91	0.95	0.88	0.77

**Table 8:** Experimental results for MIT-BIH malignant ventricular ectopy database (VF: TD2-B-lead II)

	LSTM	GRU	RNN	CNN	RSCNN
accuracy, %	93.86	90.16	94.18	88.67	80.32
sensitivity	0.94	0.90	0.94	0.89	0.80
F1_score	0.97	0.95	0.97	0.94	0.89
specificity	0.94	0.90	0.94	0.88	0.80

The next evaluation is done using tachycardia data set three (ST: TD3) known as ST. The models are evaluated against 118 samples of ST data. The model acquired sensitivities of 0.28, 0.23, 0.24, 0.19, and 0.21 for models, such as LSTM, RNN, GRU, CNN, and RSCNN, respectively, and also gained accuracies of 27.97, 22.88, 23.73, 18.64, and 21.19% for LSTM, RNN, GRU, CNN, and RSCNN, respectively. From these values, it is evident that the models were not able to detect the ST: TD3 disease. The tabulation of the results is given in Table 9. The confusion matrix for ST: TD3 is shown in the second matrix of Fig. 6. From the figure, it is apparent that only 33 samples are TP samples and the remaining samples are misclassified as TN. The performance evaluation in terms of F1 score and specificity shows the same trend as that of accuracy and sensitivity.

**Table 9:** Experimental results for ST (ST: TD3)

	LSTM	GRU	RNN	CNN	RSCNN
accuracy, %	27.97	22.88	23.73	26.50	21.19
sensitivity	0.28	0.23	0.24	0.26	0.21
F1_score	0.44	0.37	0.25	0.42	0.35
specificity	0.28	0.23	0.24	0.26	0.21

From the evaluation of the results obtained, we could analyse that the model which is trained using the AF data set is able to detect the VF ECG signal. The same model could not detect the ST signal. The reason behind the performance difference is because even though AF, VF, and ST are different kinds of tachycardia diseases there are some disease-specific features for each category. The feature-specific differences between three types of tachycardia diseases used in the present work are given in Table 10. From disease-specific features, we could analyse that the AF and VF diseases share common features of irregular heartbeat and the absence of P-wave but in the case of ST disease, it has distinct features of upright P-wave.

**Table 10:** Variation in morphology of ECG signal for different types of tachycardia diseases

Disease	P-wave	QRS complex	T-segment	Heart rate	Other features
AF	absent	not effected	not effected	>100 beats/min	presences of fibrillatory waves in the baseline
VF	absent	absent	absent	>100 beats/min	presences of irregular waves
ST	not effected	not effected	diminished	>100 beats/min	similar to normal sinus rhythm

From the analysis, we found that RNN has performed better than architectures, such as LSTM, GRU, and CNNs. The RNNs (i.e. RNN, GRU, and LSTM) and CNNs are well known for their performance in biomedical applications. The RNNs have the ability to remember the previous time step and use that information to predict the next. The various RNNs, such as RNN, LSTM, and GRU are different from each other due to the presence of gates, such as forget gate, input gate, and output gate, respectively.

The RNN is a simple feed-forward network with a feedback loop. The LSTM and GRU have additional gates to avoid long-term dependency of the previous states. In the case of tachycardia disease detection using the ECG signal, there is a possibility that the GRU and LSTM could miss the important pieces of information while passing signal vectors through different gates. In RNN, the memory cell has the ability to store all the information from the previous state, thus gaining better performance than other architectures. While considering the CNN architecture, the structural information is stored in the convolutional layer. When compared to RNNs it lacks the capacity to capture timely information. Therefore, CNN could not perform better than RNNs.

The five-fold cross-validation results for the CNN and RSCNN models trained with AF: TD1 and tested with the same are shown in Table 11. From the results, we analyse that the CNN model evaluated using five-fold cross-validation achieved approximately equal performances with that of the single fold results. In the case of RSCNN, there is a slight decrease in performance while taking an average performance of five-fold testing. From the accuracy, we observe that the RSCNN has a variation of ± 0.19%, which makes it approximately equal to the previous results achieved. Five-fold results provide a validation of experiments performed. From the results, we could analyse that the model could gain an equal amount of performance in each of the five test sets in cross-validation. This reflects that the model could learn all possible features to detect the different variations of the ECG signal.

**Table 11:** Five-fold cross-validation accuracy for CNN and RSCNN

Models	Accuracy, %	Precision	Recall	F1 score
CNN (fivefold)	95.48±0.39	0.81	0.86	0.83
CNN (singlefold)	95.32	0.851	0.781	0.815
RSCNN (fivefold)	94.96±0.19	0.83	0.78	0.80
RSCNN (singlefold)	96.08	0.92	0.91	0.91

The time-based analysis is incorporated for adding more details to the experiments. The time-based analysis includes the time taken by the model to get trained and the time taken by the model to get tested. The time required for each model to get trained with AF-TD1 data set and get tested with AF: TD1, VF: TD2-A, ST: TD3, VF: TD2-B-lead I, and VF: TD2-B-lead II data samples is shown in Table 12. The LSTM, GRU, and CNNs are trained for 1000 epochs each. For the networks, such as RNN, the model is trained for 298 epochs and RSCNN the model is trained for 105 epochs. From the experimental results, we observe that the RSCNN has taken the least time for training as the number of epochs is less, compared to all other models. Then the second least time is taken by the RNN model since it has the least number of learnable parameters among other models. Even though the GRU has the second least number of learnable parameters, it has taken much greater time than other models, while comparing other models based on accuracy and number of learnable parameters. RNN has achieved an accuracy of 96.87, 90.34, 90.88, 94.71, and 94.18% for AF: TD1 class 0, AF: TD1 class 1, VF: TD2-A, VF: TD2-B-lead II, and VF: TD2-B-lead II, respectively. RNN model has better performance than any other models in terms of accuracy and time.

**Table 12:** Training and testing time for all the deep learning models used in the present work

	LSTM	GRU	RNN	CNN	RSCNN
training time (HH.MM.SS) (AF: TD1)	08: 09:00	11: 41:54	01: 46: 09	06: 48: 51	01: 31: 04
testing time (seconds) (AF-TD1)	1.47	0.74	0.15	4.22	11.29
testing time (seconds) (VF: TD2-A)	0.52	0.43	0.23	0.31	0.71
testing time (seconds) (ST-TD3)	0.01	0.02	0.01	0.04	0.03
testing time (seconds) (VF: TD2-B-lead I)	0.05	0.03	0.03	0.11	0.23
testing time (seconds) (VF: TD2-B-lead II)	0.05	0.04	0.03	0.12	023

The comparison of the present work with respect to the current state-art-of-the method is shown in Table 13. For the AF CINC challenge data set (AF: TD1), CU VF data set (VF: TD2-A), MIT BIH Arrhythmia database (ST: TD3), and malignant ventricular ectopy database (VF: TD2-B), respectively. From the results shown in Table 13, we could interpret that the current methodology was able to achieve comparable performance with respect to the state-of-the-art methods. An exception was found in the case of ST disease. This difference in the performance of ST is because, the current methodology employs a model trained with the AF disease to detect other tachycardia diseases, such as VF and ST. The model which is trained with AF was not able to detect ST segments. The main feature of ST disease which is the upright P-waves is different from the AF and VF disease features. The features of ST do not share common feature distribution with the other tachycardia disease data set (AF and VF). The difference in feature distribution failed the model trained by the AF data set to detect ST disease.

**Table 13:** Performance comparison of the present work with respect to state-of-the-art methods for the AF: TD1, VF: TD2-A, ST: TD3, and VF: TD2-B data sets

	Accuracy	Sensitivity	F1 score	Specificity
proposed methodology (AF: TD1**)**	**96.47**	**0.93**	**0.92**	**0.90**
Chanthercrob *et al.* [39] (AF: TD1)	—	0.97	—	1.00
Gopika *et al.* [31] (AF: TD1)	96.47	0.93	0.92	0.90
proposed methodology (VF: TD2-A)	**90.88**	**0.91**	**0.95**	**0.90**
Boreiko [40] (VF: TD2-A)	—	0.83	—	—
Sohail *et al.* [41] (VF: TD2-A, VF: TD2-B)	98.03%	—	—	—
proposed methodology (ST: TD3)	**27.97**	**0.28**	**0.44**	**0.24**
Gopika *et al.* [31] MIT arrhythmia (N, S, V, F, Q)	97.94	0.98	—	—
proposed methodology (VF: TD2-B-lead I)	**94.71**	**0.95**	**0.97**	**0.95**
Proposed Methodology (VF: TD2-B-lead II)	**94.18**	**0.94**	**0.97**	**0.94**
Mohanty *et al.* [42] (VF: TD2-A/VF: TD2-B)	99.18	0.97	—	0.99
Andreotti *et al.* [24] (AF: TD1)	—	—	0.83	—
Hannun *et al.* [26] (AF: TD1)	—	—	0.837	—
Andersen *et al.* [25] (AF: TD1, VF: TD2, VT, AF, etc.)	—	0.86	—	0.98
Shashikumar *et al.* [27] (AF: TD1)	AUC-94%	—	—	—

Validation of using the feature extracted samples instead of using the raw ECG signal is done by evaluating the model performance by giving the input as raw ECG signal and the feature extracted segments. The comparison of the results acquired by feeding the model with and without feature extracted ECG signals is shown in Table 14. From the results, we could see that the RSCNN architecture has gained an accuracy of 96.08% with feature extracted signal. In the case of the RSCNN model using keeping the hyperparameters constant when fed with raw ECG signal, it could acquire only 87%. A similar trend can be seen in the case of other models also. For raw signal, the GRU model could acquire only 83.29%, but when fed with a feature extracted signal it could achieve an accuracy of 96.47%. From the results, we can analyse that by keeping model parameters constant the feature extracted segments give better performances than raw ECG signal. The feature extracted segments give a closed platform for learning the features. This helps the model to give better performance.

**Table 14:** Performance comparison of the RNN, LSTM, GRU, CNN, and RSCNN models using raw ECG signal and the handcrafted feature segments in terms of accuracy (%)

	LSTM	GRU	RNN	CNN	RSCNN
AF CINC 2017 data set (AF: TD1) featured extracted ECG signal	94.76%	96.47%	96.01%	95.32%	96.08%
AF CINC 2017 data set (AF: TD1) raw ECG signal	83.04%	83.29%	87.0%	85.49%	87%

In order to check the noise robustness, the model is tested with the noisy segments from the AF CINC challenge data set. These noisy segments are not annotated. The results are tabulated in Table 15. All the deep learning models classify the majority of the noisy segments into the normal class. The expected result was the reverse, as the noisy segments are considered the deviation from the normal class. This enforces the direction of training the deep learning algorithms to detect multi-class with the inclusion of unlabelled noisy segments along with the normal and abnormal. This can be considered as the future scope of the present work.

**Table 15:** Performance of the deep learning models towards the noisy signal in AF: TD1 data set

	LSTM	GRU	RNN	CNN	RSCNN
percentage of noisy signal falling into normal class	78.66%	78.76%	72.99%	89.17%	73.35%
percentage of noisy signal falling into abnormal class	21.34%	21.24%	27.01%	10.83%	26.65%

From the experimental results and analysis, we observed that the model trained with AF was able to detect VF and failed to detect ST. The expected results were that, if the model has learned the fast beat rhythm, it must be able to detect the other types of tachycardia diseases. From this, we were able to interpret that, even though AF, VF, and ST fall under the common disease type called tachycardia, the features learned by the model were common to AF and VF which was not applicable to ST. The presence of upright P-wave is the characteristics of an ECG signal specific to ST disease. This explains that the model just did not capture the fast beat rhythm, which is the coarse level feature to detect all the three different types of tachycardia diseases. Instead, it captured the disease specific feature, which differentiates the ST from AF and VF. This analysis from the experiments conducted led to ‘Explainable AI’.

The source codes for the experiments done in the proposed work are given in https://github.com/Sanjanakaladharan/Explainable-AI-for-Heart-Rate-Variability-in-ECG-Signal

The findings based on the experiments conducted and the results obtained are given below:

- Among all the benchmark deep learning architectures implemented for the different tachycardia disease detection, RNN was able to perform better based on our proposed transfer learning approach.
- Even though RNN was able to detect two types of tachycardia diseases namely AF and VF, it failed to detect ST. This may be due to the absence of P-wave characteristics in the trained model (using AF: TD1). ST has an up-right distinct P-wave that differentiates it from atrial and VF.

## 5 Conclusion

In this work, we proposed the transfer learning approach to interpret the features learned by the model to detect different types of tachycardia diseases. This is attained by training the five different deep learning architectures with one of the tachycardia diseases (AF: TD1). The features learned by the model using one of the tachycardia diseases are tested with all other types of tachycardia diseases, namely VF and ST. The experimental results and analysis have shown that the RNN model performed better than other standard deep learning models, such as LSTM, GRU, CNN, and RSCNN. The model was able to detect the atrial (AF: TD1) and ventricular type of tachycardia diseases (VF: TD2-A and VF: TD2-B) but failed in the case of ST (TD3). In the case of AF and VF, it is the absence of P-wave and the presence of fibrillatory waves are the features that enabled the model to detect diseases distinct from the ST. The characteristic feature for ST is the upright P-wave, which the model failed to capture when trained with one of the types of tachycardia diseases called AF. Thus, the present work led to ‘Explainable AI’, which interprets the model used to detect different types of tachycardia diseases, which have fast beat rhythm as a common characteristic of the input ECG signals.
